# First example of stepwise, zwitterionic mechanism for bicyclo[2.2.1]hept-5-ene (norbornene) formation process catalyzed by the 1-butyl-3-methylimidazolium cations

**DOI:** 10.1007/s00706-016-1735-5

**Published:** 2016-03-30

**Authors:** Radomir Jasiński

**Affiliations:** Institute of Organic Chemistry and Technology, Cracow University of Technology, Cracow, Poland

**Keywords:** Diels–Alder reaction, Nitroalkenes, Mechanism, DFT study

## Abstract

**Abstract:**

B3LYP/6-31++G(d) calculations indicated that the reaction of (2*E*)-3-phenyl-2-nitroprop-2-enenitrile with cyclopentadiene catalyzed by cations of 1,3-dialkylimidazolium ionic liquid shall not take place according to the classical scheme of one-step [2+4] Diels–Alder cycloaddition. Along the path finally leading to bicyclo[2.2.1]hept-5-ene (norbornene) with a nitro group in *endo* orientation, the process of bicarbocyclic skeleton formation shall take place according to the domino mechanism, via [2+4] heterocycloadduct. On the other hand, along the path leading finally to bicyclo[2.2.1]hept-5-ene with a nitro group in *exo* orientation, acyclic adduct with a zwitterionic nature is formed in the first reaction, which undergoes cyclisation next in the second step of the reaction.

**Graphical abstract:**

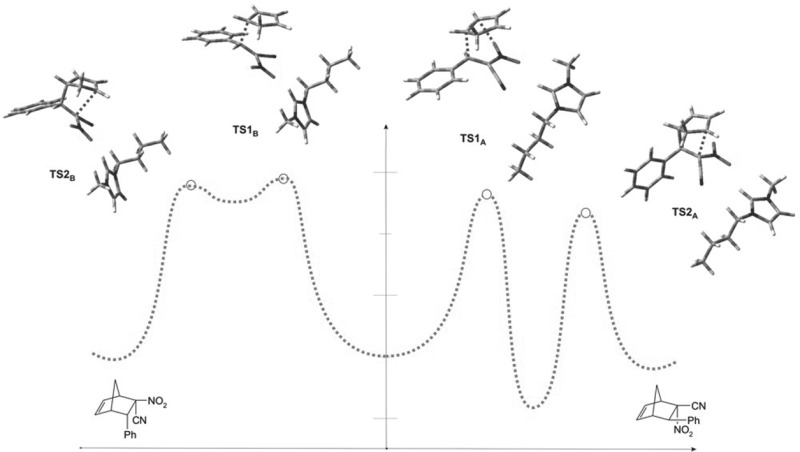

## Introduction

The Diels–Alder reaction (DA) is generally the most universal synthesis method of bicyclic carbon skeletons [1–3]. In particular, using cyclopentadiene (Cp) and ethylene derivatives, it is possible to synthesize a wide range of compounds from the bicyclo[2.2.1]hept-5-ene (norbornene) group. In general, it is accepted that DA reactions take place according to a one-step mechanism [1, 4, 5]. However, it has been shown recently [6–8] that in case of components with a high electrophilic and nucleophilic activation, the one-step mechanism may compete with a two-step, zwitterionic mechanism.

This work is a continuation of systematic studies on mechanistic aspects of DA involving conjugated nitroalkenes [9–14]. It was shown previously [11, 12], that DA occurring between (2*E*)-3-aryl-2-nitroprop-2-enenitriles **1a**–**1f** and Cp (**2**) take place under mild conditions and lead, with high yields, to mixtures of expected, stereoisomeric, nitrosubstituted bicyclo[2.2.1]hept-5-enes (Scheme 1). It should be noted also, that under similar conditions described recently, DA proceeds between the same nitroalkenes and 2,3-dimethyl-1,3-butadiene [15].

**Figure:**
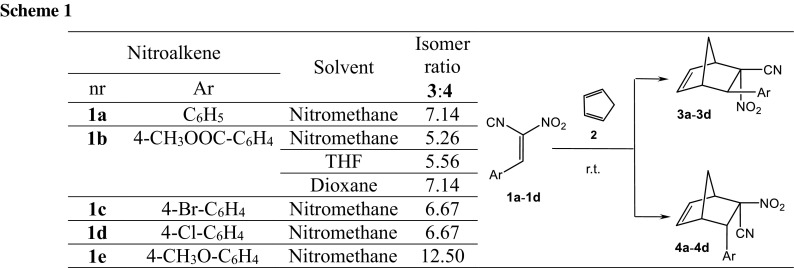

In addition, detailed quantum-chemical studies showed [13] that DA **1a**–**1f+2** take place according to a polar [16] one-step mechanism. The polar nature of these processes was also confirmed using kinetic tests [14]. Taking into account the strongly asynchronous and polar nature of transition states (TSs) of these reactions, it can be assumed that in presence of ionic liquids, it could be possible to force a two-step, zwitterionic mechanism of bicyclo[2.2.1]hept-5-ene rings creation(Scheme 2). This mechanism would be facilitated by (a) the effect of nitroalkene complexation by the cation of the ionic liquid (which should increase the global electrophilicity of the ethylene derivative [17] and stimulate delocalisation of the charges developed along the reaction) and additionally by (b) a polar environment. This type of radical change of the reaction mechanism determined by the presence of 1,3-dialkylimidazolium cations of ionic liquids is suggested in recent works on 1,3-dipolar cycloaddition of (*Z*)-*C*-methoxyphenyl-*N*-phenylnitrone to 1-chloro-1-nitroethene [18]. It should be noted that no cases of stepwise DA reactions catalyzed by ionic liquids have been presented in the literature.

Taking the above into account, within this work, DFT simulations were performed for competitive paths of the model reaction between nitroalkene **1a** linked by 1-butyl-3-methylimidazolium cation (BMIM) (currently popular and widely present in various ionic liquids used as media for cycloaddition reactions [19, 20]) and Cp (**2**). These calculations should help with understanding the role of ionic liquids in the medium of polar DA reactions.

## Results and discussion

As the DFT calculations showed, formation of a prereaction complex between nitroalkene **1a** and the BMIM cation results in lowering the enthalpy of the system by 4 kJ/mol. The formed structure has a strongly electrophilic nature: using the Domingo scale [17], complex [**1a** + BMIM] should be treated as a strong electrophile. The global electrophilicity of [**1a** + BMIM] exceeds 3.8 eV and is higher than that of highly reactive nitroalkenes such as 1,1-dinitroethene or 2-nitroprop-1-ene (see Table 1). On the other hand, Cp has a very weak electrophilic nature and in its reaction with [**1a** + BMIM] it will act as a strong nucleophile [21] (*N* > 3 eV). The high electrophilic character of complex [**1a** + BMIM] together with the strong nucleophilic character of Cp is significant, which indicates the analyzed process should be classified as a clearly polar reaction.

**Table 1:** Global electronic properties of reaction components as well as selected conjugated nitroalkenes [13, 22–24]

	*μ*/eV	*η*/eV	*ω*/eV	*Ν*/eV
**2**	−3.01	5.49	0.83	3.36
**1a**	−5.28	4.07	3.42	1.81
[**1a** + BMIM]	−5.44	3.83	3.87	1.76
1,1-Dinitroethene	−5.98	5.03	3.56	0.62
2-Nitroprop-1-ene	−5.16	5.48	2.43	1.22
(*E*)-3,3,3-Trichloro-1-nitroprop-1-ene	−5.93	5.13	3.43	0.66

**Fig. 1:**
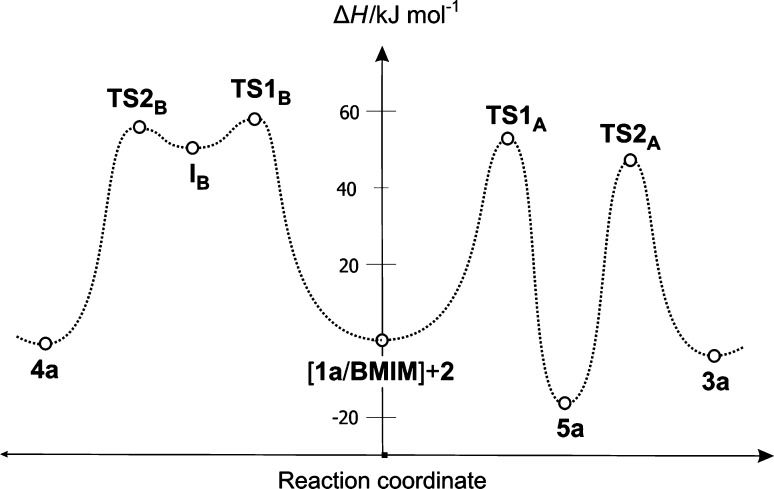
Enthalpy profiles for reactions between (2*E*)-3-phenyl-2-nitroprop-2-enenitrile (**1a**) and Cp (**2**) catalyzed by the 1-butyl-3-methylimidazolium ionic liquid cations according to B3LYP/6-31++G(d) calculations

**Fig. 2:**
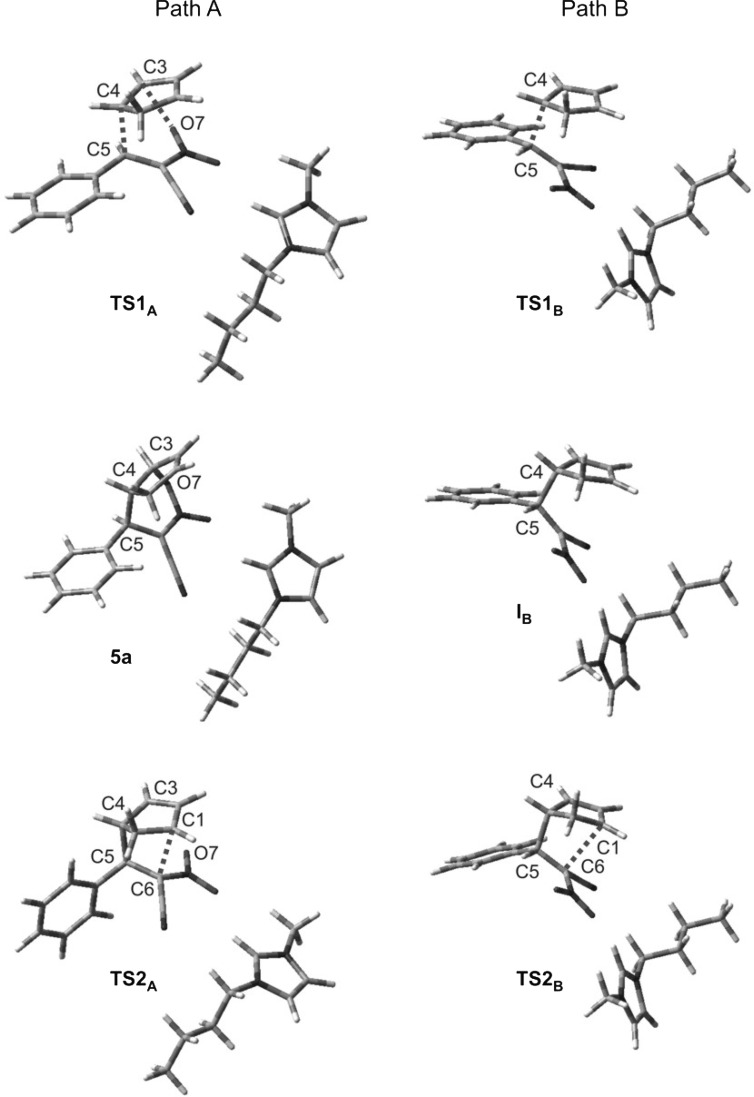
Key structures of reactions between (2*E*)-3-phenyl-2-nitroprop-2-enenitrile (**1a**) and Cp (**2**) catalyzed by the 1-butyl-3-methylimidazolium ionic liquid cations according to B3LYP/6-31++G(d) calculations

The more kinetically favorable reaction path is path A, eventually leading to bicyclo[2.2.1]hept-5-ene with nitro group in *endo* orientation. In the first step of this reaction, interactions between [**1a** + BMIM] and **2** lead to TS1_A_ (Table 2; Figs. 1, 2). It results in an enthalpy increase of over 55 kJ/mol. This barrier, however, is significantly smaller than shown by DFT calculations for a similar reaction taking place without dialkylimidazolium cations [13]. Thus, the effect of nitroalkene complexation by the ionic liquid actually increases its reactivity. The increase in enthalpy is accompanied by strong entropy reduction, which is typical for highly ordered structures.

**Table 2:** Kinetic and thermodynamic parameters for reactions between (2*E*)-3-phenyl-2-nitroprop-2-enenitrile (**1a**) and Cp (**2**) catalyzed by the 1-butyl-3-methylimidazolium ionic liquid cations according to B3LYP/6-31 ++G(d) calculations

Path	Transition	Δ*H*/kJ mol^−1^	Δ*S*/J mol^−1^ K^−1^
	**1a** + BMIM → [**1a**/BMIM]	−3.99	−123.77
	[**1a**/BMIM] + **2** → TS1_A_	55.14	−208.87
A	[**1a**/BMIM] + **2** → [**5a**/BMIM]	−18.52	−216.61
	[**1a**/BMIM] + **2** → TS2_A_	51.01	−225.27
	[**1a**/BMIM] + **2** → [**3a**/BMIM]	−5.05	−249.74
	[**1a**/BMIM] + **2** → TS1_B_	61.08	−188.79
B	[**1a**/BMIM] + **2** → [I_B_/BMIM]	52.86	−228.35
	[**1a**/BMIM] + **2** → TS2_B_	56.02	−219.77
	[**1a**/BMIM] + **2** → [**4a**/BMIM]	−1.44	−209.36

A C–C single bond between C4 and C5 atoms is formed after to pass TS1_A_. The bond between C1 and C6 atoms (which exists in the final bicyclo[2.2.1]hept-5-ene skeleton) is not formed during this step. Instead, a bond between the C3 atom introduced from Cp and the oxygen atom of the nitro group is formed (Table 3; Fig. 2). It should be noted that the progression of new bonds creation is definitely varied (Δl > 0.5). The asynchronicity in new bonds formation is accompanied by a strong effect of electron density shift towards the ethylene derivative substructure. This effect is quantitatively represented by the global electron density transfer (GEDT) index (0.68 e). Thus, TS1_A_ is definitively asynchronous and has polar nature. IRC calculations relate TS1_A_ to the addents valley (on one hand) and to the intermediate valley on the other. A detailed analysis of IRC trajectories showed that this process should be included (according to terminology by Domingo [25]) to the “one-step two stage cycloaddition” group.

**Table 3:** Key parameters for critical structures of reactions between (2*E*)-3-phenyl-2-nitroprop-2-enenitrile (**1a**) and Cp (**2**) catalyzed by the 1-butyl-3-methylimidazolium ionic liquid cations according to B3LYP/6-31++G(d) calculations

	C1-C6		C4-C5		C3-O7		GEDT
	*r*/Å	l^a^	*r*/Å	l^a^	*r*/Å	l^a^	/e
TS1_A_	3.237		2.021	0.704	2.791	0.141	0.68
[**5a**/BMIM]	3.744		1.559		1.501		0.67
TS2_A_	2.484	0.456	1.625		2.966		0.59
[**3a**/BMIM]	1.608		1.583		3.395		0.48
TS1_B_	3.179		2.010	0.793			0.71
[I_B_/BMIM]	3.130		1.665				0.95
TS2_B_	2.564	0.378	1.649				0.84
[**4a**/BMIM]	1.580		1.605				0.77

^a^ $l_{\text{X - Y}} = 1 - \frac{{r_{\text{X - Y}}^{\text{TS}} - r_{\text{X - Y}}^{\text{P}} }}{{r_{\text{X - Y}}^{\text{P}} }}$, where *r*_*X*–*Y*_^TS^ is the distance between the reaction centers X and Y in the transition structure and *r*_*X*–*Y*_^P^ is the same distance in the corresponding product

Conversion of the reacting system from TS1_A_ along the reaction coordinate leads to creation of the intermediate. However, this is not the expected zwitterion I_A_, but a nitronic ester **5a**. Its transformation into nitrosubstituted bicyclo[2.2.1]hept-5-ene **3a** takes place via TS2_A_. Within TS2_A_, the bond C3-O7 is ruptured, and subsequently the C6-C1 bond is formed. Electron density redistribution takes place within the six-membered cyclic system, with the bond between the C6 atom and the nitrogen atom introduced by the nitro group losing its double bond nature. This is accompanied by migration of the double bond within the five-membered ring. Formally, the analyzed process should be interpreted as a [3.3]-sigmatropic rearrangement. Similar rearrangements of other internal nitronic esters have been recently described [26–28].

In sum, the nature of transformations occurring during substrate conversion into *endo*-nitro bicyclo[2.2.1]hept-5-ene **3a** can be illustrated similarly to Scheme 3. As can be seen, instead of a typical DA reaction, the presented case is a “domino” type process, taking place via a hetero-Diels–Alder reaction, followed by a sigmatropic rearrangement.

**Figure:**
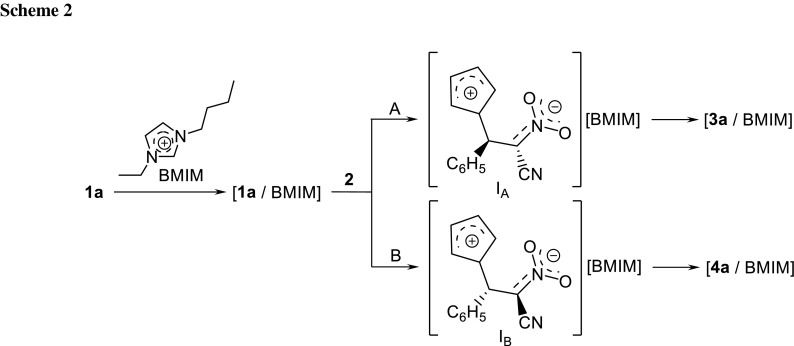

The less favorable, although not forbidden from the kinetic point of view, is path B, eventually leading to bicyclo[2.2.1]hept-5-ene with its nitro group in *exo* orientation. In this reaction, interactions between [**1a** + BMIM] and **2** lead to TS1_B_ (Table 2; Figs. 1, 2). This results in an enthalpy increase by 61.08 kJ/mol. Within TS1_B_ only one new bond is formed, namely the bond between C4 and C5 atoms. The distance between C1 and C6 atoms at this reaction stage remains outside the range typical for C–C bonds in TSs. IRC calculations relate TS1_B_ to the addent valley (on one hand) and to the intermediate valley on the other. This intermediate is the expected (Scheme 2) acyclic adduct I_B_. Its reoptimization using UB3LYP/6-31G++(d) theory level excluded diradical nature (‹S2› value is equal 0.00). At the same time, its zwitterionic nature is confirmed by the value of the GEDT index (0.95 e). I_B_ is not—under reaction conditions—a thermodynamically stable structure and easily undergoes conversion to the final product. This process takes place via TS2_B_. Within TS2_B_ the second new σ-bond is formed. In particular, it is the C1-C6 bond (Table 3). Further conversion of the reacting system along the reaction coordinate leads to nitrosubstituted bicyclo[2.2.1]hept-5-ene **4a**.

**Figure:**
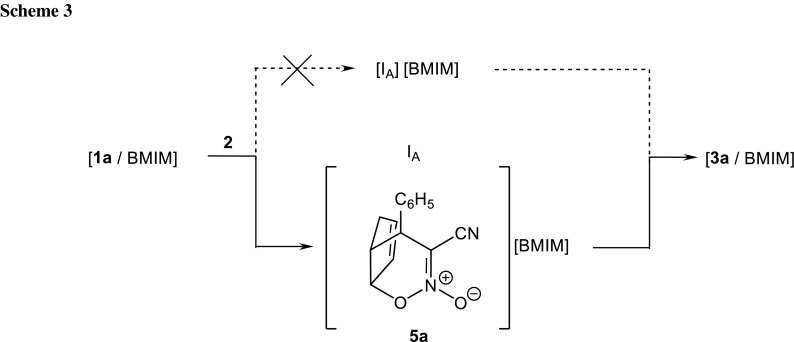

Thus, the nature of transformations occurring during substrate conversion into *exo*-nitro bicyclo[2.2.1]hept-5-ene **4a** can be illustrated as shown in Scheme 3. Formally, this process should be interpreted as a stepwise, zwitterionic cycloaddition.

## Conclusion

The reaction of (2*E*)-3-phenyl-2-nitroprop-2-enenitrile takes place under mild conditions, along two competitive paths eventually leading to stereoisomeric *endo*- and *exo*-nitro bicyclo[2.2.1]hept-5-enes. It may seem that this process takes place according to the simple, well-known DA mechanism. However, as the DFT calculations suggest, in the presence of 1,3-dialkylimidazolium cations of ionic liquids this process should take place much faster, but first and foremost, according to another, more complex mechanism. In particular, the bicyclo[2.2.1]hept-5-ene skeleton formation takes place via a discrete, zwitterionic intermediate along the path eventually leading to *exo*-nitro bicyclo[2.2.1]hept-5-ene. This is the first case for which a zwitterionic mechanism is postulated for a DA reaction catalyzed by 1,3-dialkylimidazolium cations of ionic liquids.

### Computational methods

All calculations reported in this paper were performed on “Zeus” supercomputer in the “Cyfronet” computational center in Cracow. Hybrid functional B3LYP [29] with the 6-31 ++G(d), basis set included in the GAUSSIAN 09 package [30] was used. For structure optimization of the reactants, intermediates and the reaction products the Berny algorithm was applied. First-order saddle points were localized using the QST2 [31] procedure. The TSs were verified by diagonalization of the Hessian matrix and by analysis of the intrinsic reaction coordinates (IRC) [32]. For optimized structures the thermochemical data for the temperature *T* = 298 K and pressure *p* = 1 atm were computed using vibrational analysis data. The reaction environment polarity was simulated using PCM [33]. This model has been used previously for successful diagnosis of several aspects of DA reactions involving nitroalkenes [9, 10, 13]. It was assumed that the reaction environment has dielectric constant, *ε* = 13.0, because most typical 1-butyl-3-methylimidazolium ionic liquids have *ε* ≈ 12–15 [34]. Similar approach has been successfully used by the Domingo group for the analysis of Diels–Alder reaction between *N*-tosylpyrroles and isoprene in the presence of dialkylimidazolium ionic liquids [35]. Global electron density transfer (GEDT) between substructures [36] was calculated according to the formula:

$${\text{GEDT}} = \varSigma q_{\text{A}}$$

where *q*_A_ is the net charge and the sum is taken over all the atoms of Cp.

Global electronic properties of reactants were estimated according to the equations recommended by Parr and Domingo [17, 37] using (according to Domingo suggestions [17, 36]) B3LYP/6-31G(d) theoretical level. In particular, the electronic chemical potentials (*μ*) and chemical hardness (*η*) were evaluated in terms of one-electron energies of FMO (*Ε*_HOMO_ and *Ε*_LUΜΟ_) using the following equations:

$$\mu \approx \left( {E_{{{\rm H}{\rm O}{\rm M}{\rm O}}} + E_{\text{LUMO}} } \right)/2\quad {\text{and}}\quad \eta \approx E_{\text{LUMO}} - E_{\text{HOMO}}$$

Next, the values of *μ* and *η* were then used for the calculation of global electrophilicity (*ω*) according to the formula:

$$\omega = \mu^{ 2} / 2\eta$$
